# Guy's Hospital

**Published:** 1916-09-30

**Authors:** 


### PATRIOTISM IN BRITISH HOSPITALS.

### Guy's Hospital.

Guy's has given with both hands freely to the common cause. On the one hand, many members of the hospital and teaching staffs are on active service or hold commissions in connection with the war. On the other hand, much new and valuable work has been carried out by which the labours of the diminished staff have been largely increased. Both by those who have gone and by those who remain the honour of Guy's has not merely been maintained, but fresh lustre has been shed on the old foundation.

### The Medical School.

The following members of the teaching staff of the school have received leave of absence on account of the war: Sir Alfred Fripp, and Doctors French, Hertz, Hunt, Clark, and Johnson; Messrs. Turner, Davies-Colley, Eason, Layton, Mullally, Ozanne, Schlesinger, Heaton, McNair, Dowsett, Fry, Stranack, Price-Harris, and Pritchard. In every case before their departure satisfactory arrangements were made to carry on their duties. The school is indebted to Drs. Poulton, Pembrey, Laidlaw, Nicholson, and Ryffel, and Messrs. Bellingham Smith, Mollison, Trethowan, and Doubleday for voluntarily undertaking much additional teaching, while Messrs. F. D. Saner, Zamora, Tomes, Messenger, and Wright were appointed to act temporarily as teachers in the school. Mr. Symonds, formerly lecturer on surgery in the school, undertook at short notice to be responsible for a series of clinical lectures and demonstrations in the absence of Sir Alfred Fripp. Thus the work of the school has continued without the abandonment of a single course of lectures or demonstrations for undergraduates, although, almost without exception, the members of the clinical staff have undertaken service in the various hospitals for the wounded in and round London, and although most of the physicians and surgeons hold commissions in the Royal Army Medical Corps, T.F.

About ninety medical students and eighty-five dental students are away from the hospital on military service. Nearly all of the medical students who remain are enrolled in the hospital contingent of the O.T.C., unless under military age or Colonials. All the dental students who remain, with the above exceptions, have attested, and have been temporarily exempted by the Local Tribunal. Exemptions have also been granted to medical students in the clinical period (ward work) as from April 1, 1916, and to dental students who expect to qualify this year to December 31, 1916.

It is impossible to estimate the number of Guy's men serving in the Navy and Army, but there must be many hundreds. A Roll of Service is being compiled, and will be published at a later date by *Guy's Hospital Gazette*. Of the actual hospital staff twenty members altogether are away on war duty. It is interesting to note that thirty-four Guy's men have been mentioned in dispatches. Five have been awarded the D.S.O., three the Military Cross, two have been made C.B., and one C.M.G. Among those who received the D.S.O. is Captain Lauder, one of the heroes of Wittenberg.

### The Roll of Honour.

Among the Guy's men killed in action are Lieutenants H. L. Hopkins, A. E. F. Kynaston, Montagu Pern, J. S. H. James, J. E. Rhys Evans, F. Augustus Lowe, W. R. Pryn, Edgar Faulks, Malcolm Ball, and E. F. Llarena; Second-Lieutenants L. P. Waghorn, G. B. Monk, H. B. Neely, A. C. Clifford, W. A. Stanwell, S. J. Peatfield.

Captain Malcolm Leckie, D.S.O., R.A.M.C., was one of the first to lay down his life in the care of the wounded. He was wounded at Mons on the first day of the retreat, August 24, 1914, when his "fearless and reckless devotion" under fire was conspicuous. Although partly paralysed by a bullet which penetrated to the spine, he continued to minister to cases which were brought to his bedside in the hospital at the village of Frameries, to which he was carried, and where he died shortly after.

Among distinguished Guy's students killed in action may be mentioned William Denis Browne, who was one of the singularly interesting group who gathered round Rupert Brooke. Like his friend, he joined the Royal Naval Reserve and took part in the Antwerp expedition. He was killed at the Dardanelles, June 7, 1915, leaving behind him an already assured reputation as a musician.

### Treatment of the Wounded.

A special section of the hospital for the treatment of wounded officers was opened November 2, 1914, and has admitted 506 officers to date. They have come from many different theatres of the war, British East Africa, South-West Africa, Singapore, Somaliland, Egypt, Gallipoli, and France, and there have been Canadians, Australians, and officers from the Indian Army, as well as from British regiments. Lieut. W. Kelsey, a Guy's man who obtained the Military Cross, was a patient in the officers' section.

About 100 wounded and sick soldiers from the Expeditionary Forces have been admitted to the ordinary wards, most of them requiring special treatment, as, for instance, fractured-jaw cases needing a combination of surgical and dental skill. In addition, over 1,000 recruits who required operative treatment to fit them for the Army have been admitted to Evelyn Ward, which has been specially set apart for this class of case since October, 1914. Nearly 3,000 recruits and men from the Home stations have been afforded relief in the dental out-patient, the ophthalmic, and other out-patient departments.

